# The Population Genetics of *Alternaria tenuissima* in Four Regions of China as Determined by Microsatellite Markers Obtained by Transcriptome Sequencing

**DOI:** 10.3389/fmicb.2018.02904

**Published:** 2018-12-03

**Authors:** Naibo Yang, Guoping Ma, Kenxi Chen, Xuehong Wu

**Affiliations:** College of Plant Protection, Department of Plant Pathology, China Agricultural University, Beijing, China

**Keywords:** *Alternaria tenuissima*, SSR marker, tomato, population genetic structure, next-generation sequencing

## Abstract

A total of 32,284 unigenes were obtained from the transcriptome of *Alternaria tenuissima*, a pathogenic fungus causing foliar disease in tomato, using next-generation sequencing (NGS) technology. In total, 24,670 unigenes were annotated using five databases, including NCBI non-redundant protein, Swiss-Prot, euKaryotic Orthologous Groups, Kyoto Encyclopedia of Genes and Genomes, and the Gene Ontology. A total of 1,140 simple sequence repeats were also identified for use as molecular markers. Sixteen of the simple sequence repeat loci were selected to study the population structure of *A. tenuissima*. A population genetic analysis of 191 *A. tenuissima* isolates, sampled from four geographic regions in China, indicated that *A. tenuissima* had a high level of genetic diversity, and that the selected simple sequence repeat markers could reliably capture the genetic variation. The null hypothesis of random mating was rejected for all four geographic regions in China. Isolation by distance was observed for the entire data set, but not within clusters, which is indicative of barriers to gene flow among geographic regions. The analyses of Bayesian and principal coordinates, however, did not separate four geographic regions into four separate genetic clusters. The different levels of historical migration rates suggest that isolation by distance did not represent a major biological obstacle to the spread of *A. tenuissima*. The potential epidemic spread of *A. tenuissima* in China may occur through the transport of plant products or other factors. The presented results provide a basis for a comprehensive understanding of the population genetics of *A. tenuissima* in China.

## Introduction

*Alternaria tenuissima* is an important global pathogen on a large variety of economically important crops, including broad bean, tomato, sunflower, potato, watermelon, and muskmelon (Rahman et al., 2002; Agamy et al., 2013; Wang et al., 2014; Zheng et al., 2015; Zhao et al., 2016a,b). The pathogen affects the above-ground parts of the crops, and is the causal agent of early blight, stem canker, and some fruit rots (Abdelfattah et al., 2016; Bessadat et al., 2017). Foliar diseases outbreaks caused by *A. tenuissima* are primarily epidemic and are especially devastating on tomato leaves (Agamy et al., 2013; Bessadat et al., 2017). High humidity and fairly high temperatures can lead to severe epidemics in tomato-growing regions (Bessadat et al., 2017). Although environmental conditions vary significantly in different crop production regions, once established, the infection spreads rapidly. In fact, sporadic epidemic transmission is the main factor responsible for the high frequency of occurrence of this disease (Agamy et al., 2013; Meng et al., 2015). The increasing frequency of *A. tenuissima* outbreaks has affected the distribution of *Alternaria* species responsible for causing foliar diseases (Wang et al., 2014; Zheng et al., 2015; Zhao et al., 2016a,b). The extent of genetic variation and spatial distribution in *A. tenuissima* associated with tomato foliar diseases in China, however, remains largely unknown.

Genetic variation in a species results from evolutionary events, including drift, migration, type of mating system, selection, and mutation, all of which are influenced by human activity and natural events (Wright et al., 2004; Zhan and McDonald, 2013). For example, indiscriminate use of fungicides in agro-ecosystems can increase the rate of mutation and impact the virulence and aggressiveness of a pathogen (Piotrowska et al., 2016). The artificial dissemination of a pathogen also affects migration or selection in pathogen populations and causes a change in natural ecosystems. Emergence of a sexual stage also plays an important role in dispersion (Artero et al., 2016). Species are expected to exhibit a greater level of genetic variation in response to environmental changes that increase their ability to adapt (McDonald and Linde, 2002; Meng et al., 2015). The genetic structure of a species determines the evolutionary potential of pathogen as it reflects the level of allelic diversity available to selection pressures (McDonald, 1997; Parker and Gilbert, 2004). A comprehensive understanding of the genetic structure of a species of pathogen is essential for managing disease occurrence and developing sustainable management practices (Miller et al., 2003).

Molecular markers are a reliable tool for assessing genetic variation and inferring mating systems in fungal populations (Santha Lakshmi Prasad et al., 2009; Stewart et al., 2011). To date, however, relatively few molecular markers, such as random amplified polymorphic DNA (RAPD) and amplified fragment length polymorphism (AFLP), have been reported for *Alternaria* spp. (Morris et al., 2000; Gannibal et al., 2007). Additionally, most molecular markers do not capture genetic structure with the degree of resolution and reliability that is provided by simple sequence repeats (SSRs) or microsatellites. SSRs are tandem repeat motifs of 1–6 bases that are abundantly spread throughout eukaryotic genomes and reflect genetic diversity (Andeden et al., 2015).

Genetic SSRs occur in the coding and regulatory regions of genes (Zheng et al., 2013), while genomic SSRs are in non-coding regions of the genome. Genetic SSRs have the advantage of being more highly conserved and thus more transferable across species (Chen et al., 2015). Although the identification of genetic SSRs is less expensive and time-consuming than identifying genomic SSRs, little information is available on genetic SSR markers in *A. tenuissima*. Due to their utility, it would be useful to identify SSR loci in *A. tenuissima* from transcriptomic data and design primer pairs that could be used to identify these SSR loci in genetic analyses of *A. tenuissima*.

A high level of genetic variation has been reported in *A. alternata, A. brassicicola*, and *A. solani* suggesting that a cryptic sexual stage dominates in these *Alternaria* species (Morris et al., 2000; Bock et al., 2005; Meng et al., 2015). High levels of genetic variation and evidence of sexual reproduction has also been reported in *A. tenuissima* isolated from wheat in Russia (Gannibal et al., 2007). Little information is available, however, on the population structure of *A. tenuissima* in tomato. Therefore, in the present study, a transcriptome of *A. tenuissima* was sequenced using next-generation sequencing technology (NGS) and used to identify large numbers of SSRs. This was done to determine the level of genetic diversity in *A. tenuissima* populations in China and infer the main evolutionary factors influencing the epidemic outbreaks of *A. tenuissima*. The distribution of SSR motifs in the transcriptome of *A. tenuissima* was characterized and the assembled unigenes were functionally annotated.

## Materials and Methods

### Sample Collection and Fungal Populations

**FIGURE 1:**
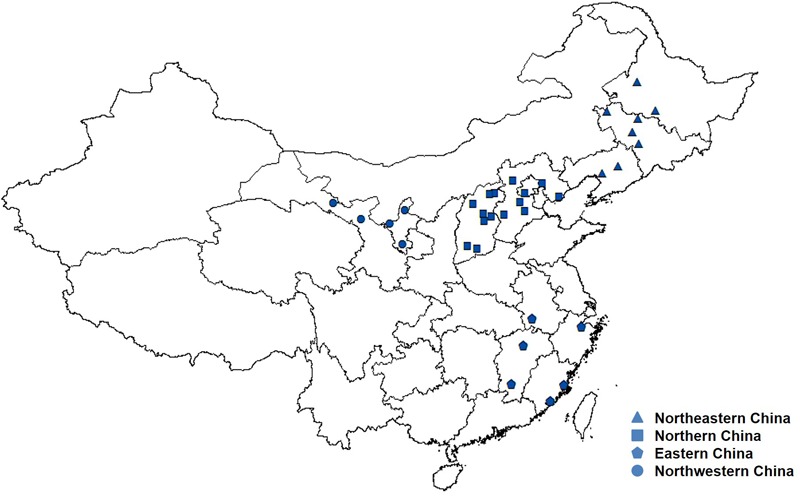
Geographic locations of the four tomato cropping regions of *Alternaria tenuissima* used in the study.

A total of 191 *A. tenuissima* isolates were collected during 2015 and 2017 from 34 sampling locations in China (Supplementary Table S1). The isolates were obtained from tomato leaves exhibiting typical symptoms of foliar disease. The 34 sampling locations from twelve provinces, autonomous region, or municipality were organized into four tomato cropping regions based on geography, climate, and agricultural management (Bernardes-de-Assis et al., 2009). They were designated Northeastern China (Heilongjiang, Jilin, and Liaoning Provinces), Northern China (Hebei, Shanxi Provinces, and Beijing Municipality), Eastern China (Anhui, Fujian, Jiangxi, and Zhejiang Provinces), and Northwestern China (Ningxia Hui Autonomous Region and Gansu Province). These groupings represent the four major tomato-cropping regions in China (Figure 1). The four geographic regions are separated from each other by more than 500 km.

Isolates were identified using the standard procedures reported in our previous study (Zheng et al., 2015). The procedure includes both morphological characteristics and molecular analyses. The collected isolates were transferred to potato carrot agar (PCA) plates and grown for 7 days at 25°C with 8 h light/16 h dark photoperiod to characterize their growth and conidia morphology. Genomic DNA was extracted from the *A. tenuissima* isolates using a cetyltrimethylammonium bromide (CTAB) procedure and used for molecular identification and additional SSR assays (Lee and Taylor, 1990). For the molecular analysis, partial coding sequences of the histone 3 gene and the internal transcribed spacer (ITS) region of ribosomal DNA (rDNA) were amplified from the extracted genomic DNA using the primer sets H3-1a/H3-1b and ITS1/ITS4, respectively (Glass and Donaldson, 1995). The PCR amplification products were shipped to Beijing TSINGKE Biotechnology Co. Ltd. (Beijing, China) for sequencing. The obtained sequence data were used to conduct BLAST searches using BLASTn on the NCBI website^1^ to identify the *Alternaria* species.

### cDNA Library Construction and Illumina Sequencing

One isolate “BJ319-1” was randomly selected from among the 191 collected *A. tenuissima* isolates. The mycelia from a culture of “BJ319-1” growing on potato dextrose agar (PDA) plates for 7 days, were harvested for the isolation of total RNA and subsequent transcriptome analysis. Total RNA was extracted using TRIzol reagent (Ambion, Thermo Fisher Scientific, United States). Any traces of DNA were then removed from the RNA extracts using DNase I (TaKaRa, Japan). The purity of the RNA extract was determined using a Nano-Drop 2000 (Thermo Fisher Scientific, United States). Qubit 2.0 (Life Technologies, United States) and an Agilent 2100 Bioanalyzer (Agilent Technologies, United States) were used to estimate the concentration and integrity of the total RNA. The cDNA library of pooled RNA was constructed with a method described by Li et al. (2014), with minor modifications. The resulting *A. tenuissima* cDNA library was sequenced using an Illumina HiSeq 4000 sequencing platform at Beijing Biomarker Technologies Co., Ltd. (Beijing, China).

### *De novo* Assembly and Unigene Annotation

To obtain high-quality reads, raw sequences from the Illumina sequencing were filtered using Trimmomatic, a flexible read-trimming tool for Illumina NGS data (Bolger et al., 2014). After filtering out low-quality reads, the resulting clean reads were deposited into the Sequence Read Archive (SRA) database^2^, under the accession number SRP136412. Subsequently, clean reads were assembled using Trinity (Haas et al., 2013).

To annotate the *A. tenuissima* transcriptome, unigenes were searched against various databases, including NCBI non-redundant protein (Nr protein), Swiss-Prot, euKaryotic Orthologous Groups (KOG), and Kyoto Encyclopedia of Genes and Genomes (KEGG) (Altschul et al., 1997; Cameron et al., 2004). Blast2GO software (Conesa et al., 2005) was used to assign the Gene Ontology (GO) terms to the unigenes. All unigene annotations were performed using the method of Wu et al. (2014).

### Development of SSRs and Primer Design

SSR loci were identified from the *A. tenuissima* transcriptome sequence data using MISA (MIcroSAtellite identification tool) and SAMtools (Li et al., 2009). The minimum number of repeats was defined as ten for mono-nucleotide repeats, six for di-nucleotide repeats, five for tri-nucleotide repeats, and three for tetra-, penta-, and hexa-nucleotide repeats. Subsequently, SSR primers were designed using Primer Premier 5.0 software (PREMIER Biosoft International, Palo Alto, CA, United States). Based on the methodology described by Chen et al. (2015), the criteria used for designing the primers were: primer length of 16–22 bp, PCR product size of 100–300 bp, annealing temperature of 40–60°C, and GC content of 40–60%.

### SSR Assays of *A. tenuissima* Populations

To further characterize the population genetics of the *A. tenuissima* populations, primers pairs were synthesized for 16 SSRs. These SSRs were used as suitable markers for subsequent analyses based on preliminary tests (Table 1). The forward primers were separately labeled with a fluorescent dye (Dye set: FAM, ROX, TRMA, HEX; Applied TSINGKE Biotechnology Co. Ltd.) at the 5′ end. PCR amplifications were performed in a 25 μL PCR mixture that included 1 μl DNA template (100 μg mL^-1^), 9.5 μL ddH_2_O, 12.5 μL 2 × T5 Super PCR Mix (TSINGKE Biotechnology Co. Ltd.), and 1 μL each of the two primers (10 μM). The amplification was conducted in an Eppendorf Mastercycler^®^ using the following protocol: an initial denaturation step at 95°C for 5 min, followed by 35 cycles of denaturation at 94°C for 30 s; annealing at 57°C for 30 s, and extension at 72°C for 30 s; with a final extension for 5 min at 72°C. The obtained amplicons were then sequenced using an ABI 3730 DNA sequencer (Applied Biosystems).

**Table 1:** Repeat motifs (RM) in the core SSR loci sequences, primer sequences, the length of the cloned alleles (LCA), and annealing temperatures (AT) in the sixteen microsatellite loci developed from transcriptomic library of *Alternaria tenuissima.*

Locus	RM	Primer sequence (5′–3′)	LCA (bp)	AT (°C)
c10062	(AC)_6_	F: CCTTCTGCTACCTCGGTCTG	154	57
		R: GACCTTCTTCCTTGATGCCA		
c8834	(GTT)_6_	F: GGTCGGTCAGATATGCAGGT	192	57
		R: GGGGTTCTCGGTCACTAACA		
c9473	(ACG)_6_	F: CGCTCAACCACAGTAACGTC	222	57
		R: CTCATCCTCGTCGCTGTCTT		
c10524	(AG)_6_	F: TTTCCCTCCTTTCCCTCCTA	202	57
		R: CGGGATCTTGATCCGTAGAA		
c6667	(TTTC)_5_	F: TGACATCACACAGGCCATTT	238	57
		R: TGAGTTTTGAGGGGGCTCTA		
c9557	(TCG)_5_	F: TCTTCTGTCCTGGGTGTTCC	246	57
		R: GGATGAATCTCCCCAGAGGT		
c9757	(GAT)_5_	F: AGGTTGATTGCAGATGAGGG	184	57
		R: AGCGCCTAATGGATGAAATG		
c9860	(GTC)_5_	F: CGTGATGTCCTGGGATTCTT	249	57
		R: GGTGGGCCATCTACTCGTAA		
c10020	(AT)_6_	F: TGAACGCGAAAAACACACTC	195	57
		R: GCTGTTCCATGTCGACGTAA		
c6100	(CGT)_5_	F: TTTGTGATGCATCTTCTCGC	202	57
		R: CGAAGGAGGTTACACTGGGA		
c3806	(AAG)_5_	F: GCCTCTTGTAGATTGGCGAG	119	57
		R: GTACCCAGGAATGGTAGCGA		
c10756	(CT)_6_	F: CAGAAATAGGATGGCGGGTA	152	57
		R: TTTTGGCTTTCGAGCACTTT		
c12318	(C)_12_	F: AGAGGTTTGGCGTGTGAAAG	209	57
		R: ATAAAATTGTGCGTGGTCGC		
c1781	(CAG)_7_	F: TTCCAAGGTTACCCTCAACG	245	57
		R: AATCCTTTGCAATCGCTGAC		
c1611	(TG)_7_	F: TGGTAGGCTGACATGGTTGA	120	57
		R: AAACCATCATCGCATCCTCT		
c6246	(CA)_10_	F: CCCGTTGAGGCTGCTACTAC	222	57
		R: ATTCCAACAGACAAGCGAGG		

### Population Genetics

Alleles were aligned using GeneMarker v.2.2.0 software (SoftGenetics, State College, PA, United States). Sequenced fragments with an identical size originating from the same primer pair were considered as an allele. Multilocus genotypes, defined as having the same alleles at each of the single SSR loci, were detected using GenClone v.2.0 (Arnaud-Haond and Belkhir, 2007). Isolates with the same multilocus haplotype were considered as the asexual progeny of a genotype.

Genotypic diversity, gene diversity (Nei, 1973), allelic richness, and clonal fraction (CF) were used to evaluate genetic variation for each of the assigned geographical groups and the pooled geographic regions (Zhan et al., 2003). The genetic variation data, except for CF, was calculated using POPGENE v. 1.32 according to the method of Meng et al. (2015). Shannon index was computed to estimate genotypic diversity (Grünwald et al., 2003). CF, defined as the percentage of isolates resulting from asexual reproduction (Zhan et al., 2003), was calculated as 1 - (number of genotypes/number of isolates assayed). The Ewens–Watterson test was used to evaluate the selective neutrality of SSR markers (Ewens, 1972; Watterson, 1978). The index population differentiation (*F*_ST_), summary heterozygosity (*H*) from each locus (Nei, 1973; Nei and Chesser, 1983), and the test for selective neutrality were also computed by POPGENE v. 1.32 (Yeh et al., 1997).

Finally, to infer the possibility of random mating in each of the geographic regions, MULTILOCUS v. 1.3 was used to test the null hypothesis of random mating using the index of association (*I*_A_) and multilocus linkage disequilibrium values (*r*_d_) by 1,000 randomizations according to Hemmati et al. (2009). If the value of *I*_A_ and *r*_d_ are not significantly different from the expected value of 0, random mating exists in the population (Brown et al., 1980). *I*_A_ is usually dependent on the number of loci included. To supplement the use of *I*_A_, a modified statistic (*r*_d_) was used in the analysis. The proportion of compatible pairs of SSR loci (PrCompat) was also performed using MULTILOCUS v. 1.3 software (Agapow and Burt, 2001). If all the observed genotypes are explained by mutation rather than recombination, two SSR loci are compatible (Estabrook and Landrum, 1975).

### Population Structure

STRUCTURE v. 2.3.4 software (Pritchard et al., 2000) was used to analyze population structure and test for admixture. A Bayesian distinct Monte Carlo Markov Chain (MCMC) approach was implemented by STRUCTURE v. 2.3.4 using the protocol described by Tsui et al. (2012). A 100,000 burn-in period followed by 1,000,000 iterations was implemented using an admixture model, and the correlated allele frequencies for *K*-values were between 1 and 10. For each simulated cluster for *K* = 1-10, ten runs were repeated independently for consistency (Tsui et al., 2012). Structure Harvester^3^ was used to compute Δ*K* (Evanno et al., 2005) to estimate the optimal *K*-value. Replicate simulations of cluster membership (*q*-matrices) at *K* = 4 were used as input for CLUMPP_Windows v. 1.1.2 (Jakobsson and Rosenberg, 2007) using the Fullsearch algorithm, with weighted H and the G similarity statistic. Summarized cluster membership matrices (*q*-values) for both individuals and populations were then visualized using DISTRUCT v. 1.1 (Rosenberg, 2004).

Nei’s unbiased genetic distance (Nei, 1978) was calculated among all pairs of sampling populations and visualized by Principal Coordinates Analysis (PCoA) with GenALEx v. 6.5 (Peakall and Smouse, 2006).

Genetic differentiation was calculated using an analysis of molecular variance (AMOVA) with ARLEQUIN v. 3.5.2.2 (Excoffier et al., 2005). Statistical significance of φ-statistics was tested based on 1023 permutations (default). Pairwise *F*_ST_ was calculated and evaluated using a randomization test with 1000 iterations utilizing ARLEQUIN v. 3.11 (Excoffier et al., 2005).

Isolation by distance (IBD) was evaluated by assessing the correlation between pairwise geographical distance and Nei’s unbiased genetic distance (Nei, 1978) for all population pairs with the package GENEPOP in R v.3.5.1 (using Isolde) (Raymond and Rousset, 1995) using 1000 random permutations.

The possibility and rate of migration among geographic regions were tested with MIGRATE v. 3.6.11 (Beerli and Felsenstein, 1999), which uses an expansion of the coalescent theory to estimate migration rates between populations (*N_e_m*) and Θ (2*N_e_μ*), where *N*_e_ is the effective population size, *m* is the constant migration rate between population pairs, and μ is the mutation rate per generation at the locus considered. Likelihood surfaces for each parameter were estimated by simulating genealogies using MCMC approach. The computations were carried out under a Brownian motion approximation of the stepwise mutation model (SMM). The runs consisted of two replicates of 10 short chains (with 10,000 genealogies sampled) and three long chains (with 500,000 genealogies sampled), with the first 10,000 genealogies discarded. A likelihood ratio test was used to compare the likelihoods of all models (Beerli and Felsenstein, 1999).

## Results

### Illumina Sequencing Data

After stringent quality assessment, a total of 32.25 million clean reads with a GC content of 54.18 and a 95.27% Quality Score 30 (Q30) were obtained from the transcriptome sequence of *A. tenuissima* (Supplementary Table S2). Clean reads accounted for 99.72% of the total raw reads (Supplementary Figure S1). Sequencing was conducted on an Illumina HiSeq 4000 sequencing platform. Based on the clean reads, 50,992 individual transcripts were identified and 32,284 unigenes were assembled with an average length of 2,007.88 bp (N50 length of transcript = 4,172 bp, which is defined as the shortest sequence length of 50% of total contigs and is used to evaluate the quality of assembled sequences) and 1,088.76 bp (N50 length of unigene = 2,451 bp), respectively. Among the unigenes, 9,555 (29.60%) were 201 to 300 bp in length; 8,304 (25.72%) were 301 to 500 bp; 5,112 (15.83%) were 501 to 1,000 bp; 3,899 (12.08%) were 1,001 to 2,000 bp; and 5,413 (16.77%) were over 2,000 bp.

### Sequence Annotation

Collectively, 24,670 unigenes were annotated utilizing five databases, NCBI non-redundant protein (Nr protein), Swiss-Prot, euKaryotic Orthologous Groups (KOG), Kyoto Encyclopedia of Genes and Genomes (KEGG), and Gene Ontology (GO). Among the unigenes, 24,570 (99.6%) exhibited significant similarity to proteins in the Nr protein database, among which 10,562 (42.8%) were also found in the Swiss-Prot database.

**FIGURE 2:**
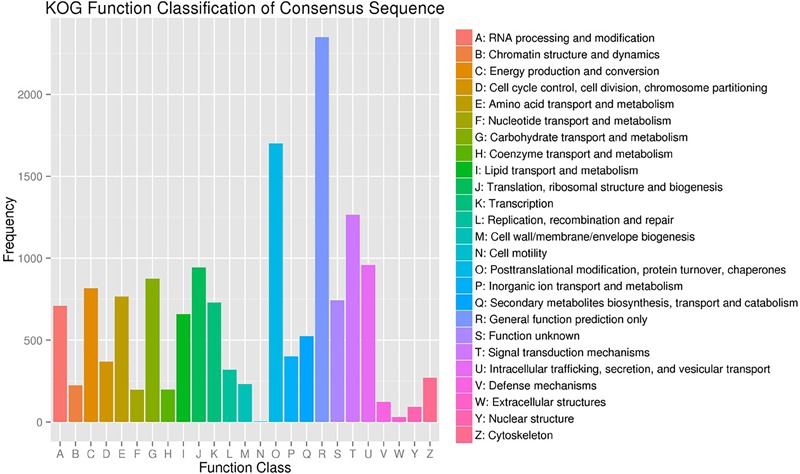
EuKaryotic Ortholog Groups (KOG) classification of assembled unigenes.

**FIGURE 3:**
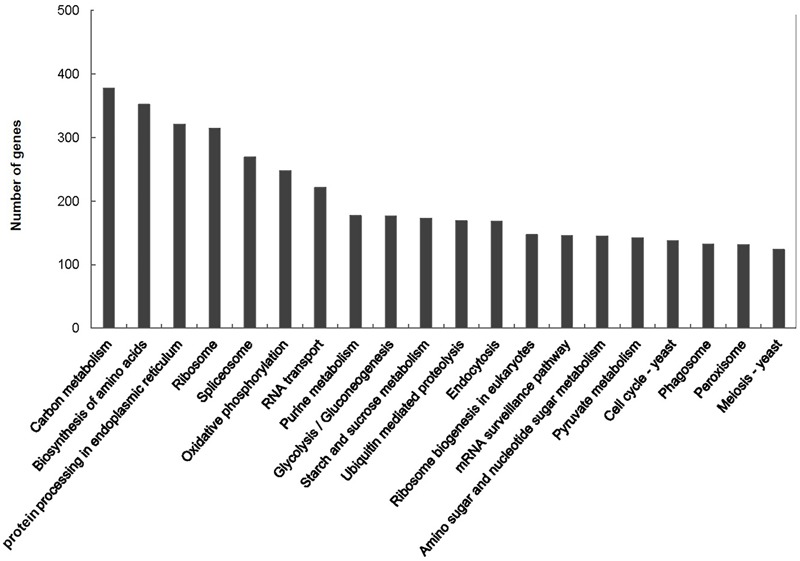
Kyoto Encyclopedia of Genes and Genomes (KEGG) classification of assembled unigenes.

**FIGURE 4:**
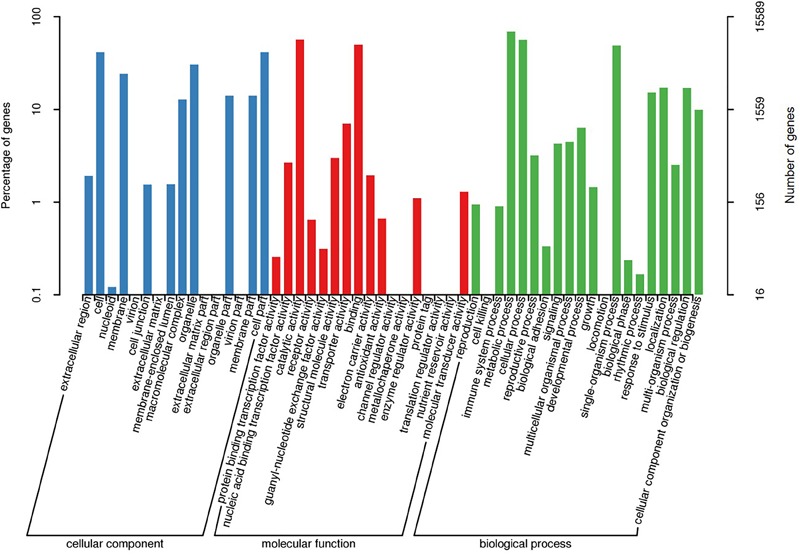
Gene ontology (GO) classification of assembled unigenes.

A total of 15,503 (62.8%) of the unigenes were classified into 25 functional categories according to the KOG functional classification (Figure 2). The general function prediction was the most highly represented category (2,349 unigenes, 15.2%). Extracellular structures (31, 0.2%), followed by cell motility (3, <0.1%), were the least represented categories. KEGG was employed to identify the biological pathways present in the transcriptome sequences obtained from *A. tenuissima*. This analysis resulted in the clustering of 7,412 (30.0%) unigenes into 111 pathways. Highly represented pathways included: carbon metabolism (378 unigenes, 5.1%), biosynthesis of amino acids (352, 4.7%), and protein processing in the endoplasmic reticulum (321, 4.3%). The top 20 biological pathways of the enriched KEGG annotations are presented in Figure 3. Further analysis indicated that 15,589 (63.2%) of the unigenes could be assigned into three GO categories: cellular component, molecular function, and biological process (Figure 4). The highest represented subcategory in the cellular component category was cell part (6,475, 41.5%). Within the molecular function category, catalytic activity (8,860, 56.8%) and binding activity (7,810, 50.1%) were the most highly represented. A total of 1,096 unigenes (7.0%) were associated with transporter activity. Under the biological process category, metabolic process (11,068, 71.0%) was the most highly represented, followed by cellular process (8,984, 57.6%), and single-organism process (7,913, 50.8%).

### Development of SSR Markers

A total of 1,140 SSRs were identified in the *A. tenuissima* transcriptome (Table 2). The SSRs were identified in 1,072 unigenes, among a total of 9,312 unigenes that were more than 1,000 bp in length. The number of repeat nucleotides in the SSRs varied from 5 to 24, with more than 10 repeats being the most abundant SSR. The percentage of motifs with 9 repeats was low (3.1%) (Table 2). A total of 111 of the unigenes contained more than one SSR. The distribution density of SSR loci in *A. tenuissima* unigenes is one per 22.9 kb and the frequency distribution of different SSR repeat numbers varies. The 487 mono-nucleotide repeat motifs were the most abundant with a frequency of 42.7%, followed by tri- (368 or 32.3%), di- (258 or 22.6%), tetra- (15 or 1.32%), penta- (7 or 0.6%), and hexa-nucleotide (5 or 0.4%) repeat motifs (Table 2). Sixteen polymorphic SSR markers were selected for population genetic structure analysis based on the presence of different motifs. The unigenes with SSR markers were annotated with 51 functions, assigned to three categories in GO terms, and grouped into 13 different classifications in the KOG database. The unigenes with SSR markers were mapped onto the 15 pathways in the KEGG pathway database (Supplementary Table S3).

**Table 2:** Summary of the number of nucleotide repeat units in *Alternaria tenuissima* SSR loci.

Repeat motif		Number of repeats							
		5	6	7	8	9	10	>10	Total
Mono-nucleotide (487)	A/T	-^a^	-	-	-	-	120	349	469
	C/G	-	-	-	-	-	5	13	18
Di-nucleotide (258)	AC/GT	-	13	7	4	3	1	6	34
	AG/CT	-	23	14	18	11	7	8	81
	AT/TA	-	10	5	2	7	1	1	26
	TG/CA	-	13	8	6	5	1	1	34
	TC/GA	-	20	19	10	7	7	8	71
	Others	-	7	1	-	-	-	4	12
Tri-nucleotide (368)	AAC/GTT	8	1	2	1	-	-	-	12
	AAG/CTT	30	7	3	1	-	-	-	41
	ACC/GGT	10	3	3	-	-	-	-	16
	AGA/TCT	14	2	5	1	-	-	-	22
	CGA/TCG	21	6	1	1				29
	GGC/TGC	20	4	2					26
	Others	130	48	33	10	1	-	-	222
Tetra-nucleotide (15)	TTGA/CACT	1	1	-	-	-	-	-	2
	AATC/TGGA	2	-	-	-	-	-	-	2
	TCCT/CTGG	-	2	-	-	-	-	-	2
	GTCA/CCTA	1	1	-	-	-	-	-	2
	Others	4	2			1			7
Penta-nucleotide (7)	CGAGG/ GCCAT	2	-	-	-	-	-	-	2
	AACCA/ GTTTT	1	1	-	-	-	-	-	2
	CCACA/ CTATT	2	-	-	-	-	-	-	2
	Others	-	1	-	-	-	-	-	1
Hexa-nucleotide (5)	CCGGTT/ TCATCC	1	1	-	-	-	-	-	2
	GAAGAC/ GATGGC	1	1	-	-	-	-	-	2
	Others	1	-	-	-	-	-	-	1
Total		249	167	103	54	35	142	390	1140
Ratio (%)		21.8%	14.6%	9.0%	4.7%	3.1%	12.5%	34.2%	100.0%

*^*a*^“-” represents no repeat unit.*

### Genetic Variation and Linkage Disequilibrium

Sixteen SSR markers were used to analyze the genetic structure of *A. tenuissima* in four geographic regions in China (Table 1). The flanking primers designed for each SSR provided distinct amplicons of the expected size. The observed fixation indexes had a 95% confidence interval for the analysis of selected neutrality of the SSR loci, suggesting that each SSR conformed to selective neutrality (Table 3). The 191 isolates from the four populations of *A. tenuissima* were determined to represent 182 distinct genotypes. Genotypic diversity was 0.87 and the CF was 0.05 in the population pooled from the four geographic regions (Table 4). Among the geographic regions, 175 were detected only once, 6 genotypes detected twice, and one genotype detected four times. A total of 180 of the genotypes were detected in only 1 geographic region, while 2 genotypes were present in two geographic regions. No unique genotypes were found to be present in three or four geographic regions. The genotypic diversity of isolates collected from Eastern China was higher than in the other three regions, Northeastern China, Northern China, and Northwestern China (Table 4).

**Table 3:** Size range, number of isolates analyzed (*n*), number of alleles (*N*_a_), and test for neutrality of the SSR loci identified in the transcriptome sequence data of *Alternaria tenuissima.*

Locus	SSR (bp)	Number of isolates	Number of alleles	Test for neutrality				
				OF^a^	Mean	*SE*^b^	L_95_^c^	U_95_^d^
c10062	147–153	179	4	0.7225	0.6007	0.0325	0.3182	0.9345
c8834	188–197	190	4	0.4901	0.6021	0.0327	0.3203	0.9383
c9473	215–224	185	4	0.5509	0.6045	0.0337	0.3142	0.9367
c10524	193–215	182	8	0.2441	0.3755	0.0189	0.1967	0.7219
c6667	225–241	190	4	0.4498	0.6101	0.0342	0.3219	0.9483
c9557	246–254	181	3	0.8659	0.6932	0.0354	0.3710	0.9781
c9757	154–184	187	4	0.4151	0.5889	0.0344	0.3213	0.9374
c9860	246–252	187	3	0.4503	0.7086	0.0337	0.3789	0.9683
c10020	191–199	190	5	0.6171	0.5264	0.0303	0.2683	0.8891
c6100	202–205	189	2	0.6059	0.8263	0.0285	0.5017	0.9895
c3806	111–192	183	13	0.4148	0.2433	0.0082	0.1340	0.4884
c10756	151–163	191	6	0.5937	0.4677	0.0278	0.2437	0.8506
c1611	121–163	181	11	0.1562	0.2777	0.0102	0.1541	0.5467
c12318	201–210	176	9	0.2443	0.3345	0.0144	0.1754	0.6386
c1781	234–246	185	5	0.4163	0.5240	0.0297	0.2674	0.8959
c6246	220–244	187	12	0.2031	0.2603	0.0094	0.1400	0.5177

*^*a*^Observed frequency of marker. ^*b*^Standard error of the mean. ^*c*^Lower 95% confidence limit. ^*d*^Upper 95% confidence limit*.

**Table 4:** Genetic diversity in four *Alternaria tenuissima* geographic regions sampled from the principle tomato production regions in China.

Population	Sample size	Number of genotypes	Clonal fraction	Diversity	Number of loci	Polymorphism	Number of alleles per locus	Number of private alleles per locus	Gene diversity
Northeastern China	44	42	0.05	0.88	16	15	3.69	0.25	0.48
Northern China	72	70	0.03	0.91	16	16	5.13	0.31	0.49
Eastern China	38	36	0.05	1.08	16	16	5.00	0.69	0.56
Northwestern China	37	36	0.03	0.87	16	16	4.00	0.25	0.48
Total	191	182	0.05	0.87	16	16	3.94	0.38	0.48

The total number of alleles in the four geographic regions ranged from 2 to 13, and the number of private alleles ranged from 0.25 to 0.69 (Table 4). *A. tenuissima* from Eastern China had the most private alleles, followed by Northeastern China, Northern China, and Northwestern China. Correspondingly, the population from Eastern China also had the highest genetic diversity value among all four populations. Values of gene diversity ranged from 0.48 in Northwestern China to 0.56 in Eastern China (Table 4). The proportion of total genetic diversity attributed to population differentiation (*F*_ST_) ranged from 0.309 to 0.582 for the sixteen SSR loci, with an overall average of 0.461. The gene diversity per locus ranged from 0.102 to 0.854 (Table 5).

**Table 5:** Summary of index population differentiation (*F_ST_*) and heterozygosity (*H*) from each locus in *Alternaria tenuissima* isolates collected from China.

Locus	*F*_ST_	*H*
c10062	0.442	0.291
c8834	0.456	0.496
c9473	0.582	0.493
c10524	0.499	0.770
c6667	0.407	0.546
c9557	0.450	0.102
c9757	0.476	0.544
c9860	0.459	0.574
c10020	0.492	0.365
c6100	0.309	0.398
c3806	0.471	0.545
c10756	0.542	0.431
c1611	0.444	0.854
c12318	0.378	0.744
c1781	0.527	0.589
c6246	0.447	0.770
Mean	0.461	

In an analysis of multilocus gametic disequilibrium, two measures of association, linkage disequilibrium (*I*_A_) and proportion of compatible pairs of loci (*r*_d_), were found to be significant in the four geographic regions for the total sample (all four geographic regions combined), indicating that the null hypothesis of complete panmixia was rejected (Table 6).

**Table 6:** Proportion of compatible pairs of loci (PrCompat), index of association (*I*_A_), and multilocus linkage disequilibrium (*r*_d_) for Chinese populations of *Alternaria tenuissima* obtained from infected tomato leaves.

Parameter	Northeastern China	Northern China	Eastern China	Northwestern China	Total
Proportion of compatible pairs of loci	0.158 (*P* = 0.150)^a^	0.142 (*P* = 0.218)	0.133 (*P* = 0.012)	0.283 (*P* = 0.168)	0.017 (*P* = 0.036)
Index of association	0.162 (*P* = 0.036)	0.222 (*P* = 0.004)	0.437 (*P* < 0.002)	0.543 (*P* < 0.002)	0.232 (*P* < 0.002)
Multilocus linkage disequilibrium	0.012 (*P* = 0.036)	0.015 (*P* = 0.004)	0.029 (*P* < 0.002)	0.037 (*P* < 0.002)	0.016 (*P* < 0.002)

*^*a*^1-tailed P-values as calculated in MULTILOCUS ver. 1.3 (Agapow and Burt, 2001) after 1,000 randomizations.*

### Population Structure and Differentiation

**FIGURE 5:**
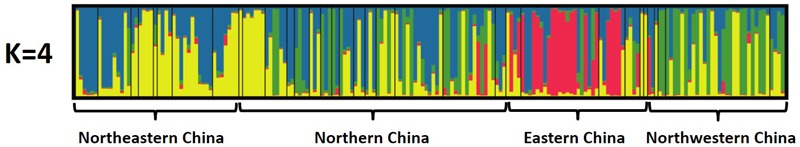
Population structure of *A. tenuissima* based on 16 microsatellites (different shadings represent different genetic groups; each column represents an individual isolate, and the height of the column segments shows the probability of assignment of this isolate to a particular genetic group. The height of each shaded region within an individual bar is the measure of proportional affiliation. When *K* = 4, *q*1 red, *q*2 green, *q*3 yellow, *q*4 blue, individuals with membership coefficients of *q*_i_ ≥ 0.7 were assigned to a specific genetic cluster).

The Bayesian cluster analysis using STRUCTURE v. 2.3.4 indicated that the number of genetically distinct ancestral populations was best represented by *K* = 4 clusters, which was the highest value of Δ*K* (Figure 5 and Supplementary Figure S2). The isolates from Northeastern China were assigned to cluster *q*4 (17 isolates, 38%) and *q*3 (15 isolates, 34%). The fifteen populations from Northern China exhibited a high level of admixture, and the isolates were assigned to cluster *q*4 (21 isolates, 29%) and *q*3 (19 isolates, 26%), followed by *q*2 (16 isolates, 22%). Most of the isolates from Eastern China were assigned to cluster *q*1 (18 isolates, 47%), and only one isolate was assigned to cluster *q*2. Most of the isolates from Northwestern China were assigned to *q*2 (16 isolates, 43%) and to a lesser extent *q*3 (8 isolates, 22%). Only two Northwestern China isolates were assigned to cluster *q*4.

**FIGURE 6:**
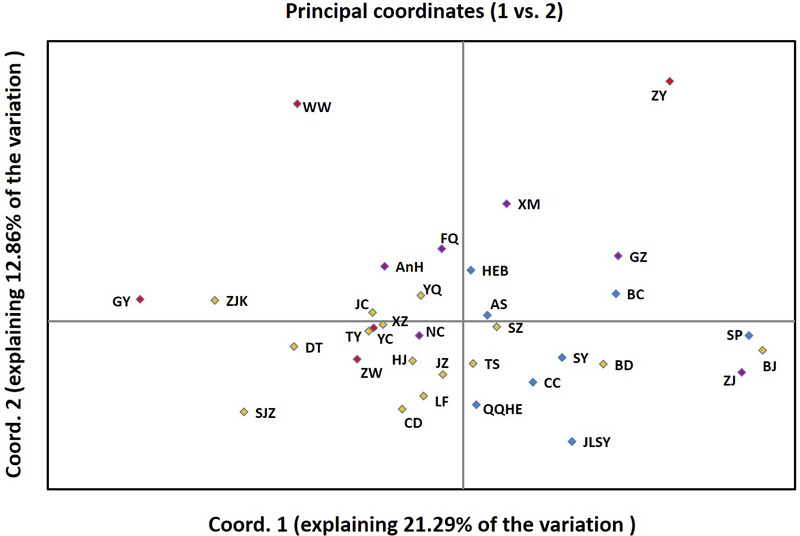
Principal Coordinates Analysis (PCoA) among 34 populations based on Nei’s genetic distance using GenAlEx.

Based on the PCoA, the eight populations from Northeastern China clustered within the two right quadrants of the first PCoA axis (Figure 6). The fifteen Northern China populations were spread across the first component space (explaining 45% of the variation), partially overlapping with Eastern China and two Northwestern China regional populations. Northwestern China and Northeastern China regional populations were slightly differentiated in the second PCoA axis (explaining 12.86% of the variation). The populations in Eastern China and Northwestern China tended to fall into different clusters in the first PCoA axis (Figure 6). The PCoA results were similar to results obtained in the STRUCTURE analysis. The genetic clusters were not completely grouped according to geographic region in the PCoA analysis, which may be explained by the populations with small sample size.

The analysis of molecular variance (AMOVA) performed on the 34 populations indicated that 13.25 and 86.75% of the genetic variation was attributed to variations among and within populations, respectively (*P* < 0.001) (Table 7). AMOVA was used to further analyze the level of differentiation among the four geographic regions established in the analysis utilizing STRUCTURE and geography. AMOVA attributed 5.43, 8.88, and 85.69% of the total variation to variations among geographic regions, among sampling locations within geographic region, and among individual isolates within populations, respectively, all of which were highly significant (*P* < 0.001) (Table 7).

**Table 7:** Analysis of molecular variance (AMOVA) for *Alternaria tenuissima* populations based on (i) sampling locations, and (ii) four geographic regions.

Source of variation	d.f.	SS	Variance	%	*P*-values
(i)					
Among all 34 sampling locations	33	198.47	0.50	13.25	<0.001
Within each of 34 sampling locations	157	515.72	3.28	86.75	<0.001
Total	190	714.19	3.79		
(ii)					
Among 4 geographic regions	3	52.03	0.21	5.43	<0.001
Among populations within 4 geographic regions	30	146.44	0.34	8.88	<0.001
Within 34 sampling locations	157	515.72	3.28	85.69	<0.001
Total	190	714.19	3.83		

*Degree of freedom (d.f.), sum of squares (SS), variance estimates, percentage of total variation (%) contributed by sampling locations, geographic regions and individual within sampling locations are presented*.

In general, pairwise genetic differentiations (*F*_ST_) between populations were not significant within the geographic regions Northern China, Eastern China, and Northwestern China, except for Baoding city (Supplementary Table S4). These results indicate that the level of genetic differentiation within the Northern China, Eastern China, and Northwestern China are similar.

The strength of the correlation was weak and non-significant within the genetic clusters, Northeastern China (*r*^2^ = 0.0151, *P* = 0.668), Northern China (*r*^2^ = 0.0580, *P* = 0.999), Eastern China (*r*^2^ = 0.0847, *P* = 0.907), and Northwestern China (*r*^2^ = 0.3949, *P* = 0.007) (Supplementary Figure S3), however, a significant correlation was observed between genetic distances *F_ST_*/(1-*F_ST_*) and geographical distances (km) for the entire data set (*r^2^* = 0.0362, *P* < 0.001). These results suggest that isolation by distance exists among the geographic regions.

Considerable levels of gene flow were observed among the geographic regions with an estimated number of migrants per generation M (*2N_e_m*) ranging from 0.54 (Eastern China from Northwestern China) to 3.70 (Northwestern China from Northern China) (Table 8). The observed gene flow was asymmetric between Eastern China and Northwestern China (1.13 vs. 0.54), depending on the direction of the gene flow.

**Table 8:** Estimates of the mean population mutation rate (2*N_e_μ*) and mean number of migrants per generation M (2*N_e_m*).

Source population	Sink population				
	Theta	Northeastern China	Northern China	Eastern China	Northwestern China
Northeastern China	0.6544	–	0.90 (0.82–0.99)	1.56 (1.44–1.69)	1.56 (1.41–1.71)
Northern China	0.7016	0.93 (0.84–1.02)	–	1.11 (0.99–1.23)	3.70 (3.47–3.94)
Eastern China	0.7859	1.27 (1.16–1.39)	1.23 (1.12–1.34)	–	1.13 (1.00–1.28)
Northwestern China	0.5152	0.82 (0.75–0.90)	1.36 (1.27–1.46)	0.54 (0.47–0.60)	–

## Discussion

Whole genome sequences in the genus *Alternaria* have been obtained for *A. consortialis* (GenBank accession no. BCGG00000000), *A. alternata* (LMXP00000000), *A. arborescens* (AIIC00000000), and *A. brassicicola* (PHFN00000000) (Hu et al., 2012; Nguyen et al., 2016). A whole genome or transcriptome sequence of *A. tenuissima*, however, has not been reported or deposited in a public DNA database. In the current study, Illumina sequencing of a transcriptome of *A. tenuissima* generated 32.25 million reads with a 95.27% Q30 and 32,284 unigenes were predicted after assembly. The N50 length of the unigenes was 2,451 bp, which was longer than the N50 obtained from the transcriptome sequencing of *Alternaria* sp. MG1 (N50 = 2,153 bp) using the Illumina HiSeq 2500 platform in a previous study (Che et al., 2016). Collectively, the results indicate that the quality and integrity of the obtained sequences are high.

Next-generation sequencing is a highly efficient and low-cost technology that can be used to develop large numbers of new SSR markers (Zhang et al., 2014). Detecting SSR markers in NGS data derived from a transcriptome is more efficient and rapid than previous, standard methodologies (Zheng et al., 2013). A total of 1,140 SSR loci were identified from 1,072 unigene sequences of *A. tenuissima.* Approximately 11.5% of the transcriptomic sequences contained SSR loci. The distribution density of SSRs in *A. tenuissima* is similar to many other higher plant species, such as rice, wheat, and soybean, which generally express a larger number of genes in a transcriptome than fungi due to the overall size of their genomes. Our results clearly identified a large number of SSR loci in the genes expressed in the *A. tenuissima* transcriptome.

Genetic markers should be selectively neutral, moderately diverse, and not linked if they are to be reliably used to study population genetics (Brown, 1996; Cooke and Lees, 2004). In the current study, sixteen SSR loci were selected from among different unigenes to analyze genetic diversity in *A. tenuissima* populations from four geographic regions. Most of the unigenes with SSR loci had different annotations in GO, KOG, and KEGG databases. The average size of a genome within different *Alternaria* species is more than 30 Mb (Hu et al., 2012; Woudenberg et al., 2015). Therefore, the probability that the sixteen selected genetic SSR markers are linked is extremely low based on the size of the genome.

Sexual recombination is expected to produce high levels of genetic diversity and random association among different loci (Milgroom, 1996; Kreis et al., 2016). In recent studies, some *Alternaria* species, such as *A. solani* (Meng et al., 2015), *A. brassicicola* (Morris et al., 2000), and *A. helianthin* (Santha Lakshmi Prasad et al., 2009) have been reported to have high levels of genetic diversity and recombination. Stewart et al. (2011), based on the results of mating system tests, suggested that *Alternaria* has a sexual cycle. Linkage equilibrium was found in *A. brassicicola* among the microsatellite loci (Linde et al., 2010). The complete sexual cycle of the above *Alternaria* species, however, has not been observed in any parts of the world. In the present study, the analysis of genotypic disequilibrium of populations from four geographic regions revealed a significant degree of non-random association, although high levels of diversity were observed. These results are consistent with Meng et al. (2015), who found that populations of *A. solani* from the Fujian Province (Eastern China) displayed high genetic variation and a lack of random mating. Bock et al. (2005) reported high levels of genetic diversity with a significant level of linkage disequilibrium in populations of *A. brassicicola* and suggested that recombination occurred only occasionally. Van Der Waals et al. (2004) indicated that the high genetic variation in *A. solani* could be accounted for by mutations rather than by sexual reproduction. These results suggest that random mating is not the main biotic factor that governs the high variation present in the four geographic regions.

High levels of genetic diversity were found to be present in the four geographic regions and within each sampled population, except for populations with a small sample size (e.g., Beijing Municipality, Songyuan, Ganzhou, Shaoxing, and Zhangye cities) (Table 4 and Supplementary Table S1). There are reports of *A. tenuissima* causing foliar diseases in China on wheat (Bensassi et al., 2009), potato (Zheng et al., 2015), and watermelon (Zhao et al., 2016a). These crops are often grown in rotation with tomato in some regions of China. The high genetic variation within each geographic region and the low spatial differentiation among different geographic regions are similar to in the findings of a study of *A. alternata* in China (Meng et al., 2018). The genetic structure of the Northeastern China, Northern China, and Northwestern China geographic regions were highly admixed and could not be separated into three single major clusters by admixture and principal coordinate analyses. These results comply with gene flow driven by anthropogenic activities occurring in geographically closer populations which exchange genetic information over time, and have a tendency to exhibit a higher genetic similarity (Meng et al., 2018).

Samples in the current study were collected from four geographic regions, separated from each other by more than 500 km. It is difficult for pathogen spores to be disseminated such a distance via the air. The isolation by distance observed for the entire data set, but not within geographic regions, is indicative of a barrier to gene flow. Seed-borne dispersal or transport of other goods contaminated with *A. tenuissima*, however, may account for the observed gene flow between the geographic regions of Northeastern China, Northern China, and Northwestern China (Malik et al., 1991; Bock et al., 2005; Meng et al., 2015). Long distance dispersal via human-mediated gene flow was also reported in populations of *A. alternata* in potato growing areas of China (Meng et al., 2018) and *Rhynchosporium secalis* in agricultural systems (Linde et al., 2009). Our present results suggest that human-mediated dispersal also plays an important role in the dynamics of the population genetic structure of *A. tenuissima*.

In contrast to Northeastern China, Northern China, and Northwestern China, the Eastern China region exhibited a relatively simple genetic structure (Figure 1). The sampled locations in the Eastern China region are geographically far from the other three tomato-cropping regions and separated from them by the Yellow and Yangtze Rivers. We suggest that the populations located within Eastern China may be separated by weak natural barriers. In this scenario, the significant correlation between genetic differentiation and geographic distance would mainly be influenced by the population genetic structure in the Eastern China geographic region. This infers that genetic isolation exists between the Eastern China geographic region and the other three tomato-cropping regions.

In recent years, *A. tenuissima* has become an important pathogen, causing foliar disease in various crops throughout China (Wang et al., 2014; Zheng et al., 2015; Zhao et al., 2016a,b). A comprehensive understanding of the population genetics of *A. tenuissima* has been lacking. In the present study, high levels of genetic diversity were determined to be present in *A. tenuissima* potentially brought about by gene flow among individuals within the populations. This may explain why *A. tenuissima* has developed the ability to infect different crops. The population genetics and biology of other tomato-growing regions in China (e.g., Central China and Southern China) have yet to be determined. Additional population genetic studies of *Alternaria* are needed for other geographic regions in China and further analyses are needed to determine the population genetic structure of *Alternaria* isolates over wider geographic regions of China.

## Data Archiving Statement

Data for this study will be available at the Dryad Digital Repository after manuscript is accepted for publication.

## Author Contributions

NY and XW conceived and designed the study. NY, GM, and KC performed the experiments. NY and XW wrote the paper. XW reviewed and edited the manuscript.

## Conflict of Interest Statement

The authors declare that the research was conducted in the absence of any commercial or financial relationships that could be construed as a potential conflict of interest.

## Footnotes

1 https://blast.ncbi.nlm.nih.gov/Blast.cgi

2 https://www.ncbi.nlm.nih.gov/sra

3 http://taylor0.biology.ucla.edu/struct_harvest/

## Supplementary Material

FIGURE S1 The percentage of clean reads, adapter related reads, and low quality reads.

FIGURE S2 The estimated Delta K (K) for number of clusters ranging from 2 to 10 in STRUCTURE analysis.

FIGURE S3 Plot of isolation by distance for the entire population, the geographic region Northern China, the geographic region Northeastern China, and the geographic region Eastern China.

TABLE S1 Isolates of *Alternaria tenuissima* comprising four different geographic regions in China.

TABLE S2 Transcriptome reads and assembled contig information for *Alternaria tenuissima*.

TABLE S3 The SSR of unigene annotations in KOG and GO database, and the pathway of KEGG orthology.

TABLE S4 Pairwise *F*_ST_ calculated with Arlequin (assessed after 1000 permutations).

## References

[B1] Abdelfattah A. Wisniewski M. Droby S. Schena L. (2016). Spatial and compositional variation in the fungal communities of organic and conventionally grown apple fruit at the consumer point-of-purchase. *Hortic. Res.* 3:16047. 10.1038/hortres.2016.47 27766161 PMC5051542

[B2] Agamy R. Alamri S. Moustafa M. F. Hashem M. (2013). Management of tomato leaf spot caused by *Alternaria tenuissima* Wiltshire using salicylic acid and Agrileen. *Int. J. Agric. Biol.* 15 266–272.

[B3] Agapow P. M. Burt A. (2001). Indices of multilocus linkage disequilibrium. *Mol. Ecol. Notes* 1 101–102. 10.1046/j.1471-8278.2000.00014.x

[B4] Altschul S. F. Madden T. L. Schäffer A. A. Zhang J. H. Zhang Z. Miller W. (1997). Gapped BLAST and PSI-BLAST: a new generation of protein database search programs. *Nucleic Acids Res.* 25 3389–3402. 10.1093/nar/25.17.3389 9254694 PMC146917

[B5] Andeden E. E. Baloch F. S. Çakır E. Toklu F. Özkan H. (2015). Development, characterization and mapping of microsatellite markers for lentil (*Lens culinaris* Medik.). *Plant Breed.* 134 589–598. 10.1111/pbr.12296

[B6] Arnaud-Haond S. Belkhir K. (2007). GENCLONE: a computer program to analyse genotypic data, test for clonality and describe spatial clonal organization. *Mol. Ecol. Notes* 7 15–17. 10.1111/j.1471-8286.2006.01522.x

[B7] Artero A. S. Silva J. Q. Albuquerque P. S. B. Bressan E. A. Leal G. A. Sebbenn A. M. (2016). Spatial genetic structure and dispersal of the cacao pathogen *Moniliophthora perniciosa* in the Brazilian Amazon. *Plant Pathol.* 66 912–923. 10.1111/ppa.12644

[B8] Beerli P. Felsenstein J. (1999). Maximum likelihood estimation of migration rates and effective population numbers in two populations using a coalescent approach. *Genetics* 152 763–773. 10353916 10.1093/genetics/152.2.763 PMC1460627

[B9] Bensassi F. Zid M. Rhouma A. Bacha H. Hajlaoui M. R. (2009). First report of *Alternaria* species associated with black point of wheat in Tunisia. *Ann. Microbiol.* 59 465–467. 10.1007/BF03175132

[B10] Bernardes-de-Assis J. Storari M. Zala M. Wang W. Jiang D. ShiDong L. (2009). Genetic structure of populations of the rice-infecting pathogen *Rhizoctonia solani* AG-1 IA from China. *Phytopathology* 99 1090–1099. 10.1094/PHYTO-99-9-1090 19671012

[B11] Bessadat N. Berruyer R. Hamon B. Bataille-Simoneau N. Benichou S. Mebrouk K. (2017). *Alternaria* species associated with early blight epidemics on tomato and other *Solanaceae* crops in northwestern Algeria. *Eur. J. Plant Pathol.* 148 181–197. 10.1007/s10658-016-1081-9

[B12] Bock C. H. Thrall P. H. Burdon J. J. (2005). Genetic structure of populations of *Alternaria brassicicola* suggests the occurrence of sexual recombination. *Mycol. Res.* 109 227–236. 10.1017/S0953756204001674 15839106

[B13] Bolger A. M. Lohse M. Usadel B. (2014). Trimmomatic: a flexible trimmer for Illumina sequence data. *Bioinformatics* 30 2114–2120. 10.1093/bioinformatics/btu170 24695404 PMC4103590

[B14] Brown A. H. D. Feldman M. W. Nevo E. (1980). Multilocus structure of natural populations of *Hordeum spontaneum*. *Genetics* 96 523–536. 17249067 10.1093/genetics/96.2.523 PMC1214315

[B15] Brown J. K. M. (1996). The choice of molecular marker methods for population genetic studies of plant pathogens. *New Phytol.* 133 183–195. 10.1111/j.1469-8137.1996.tb04353.x 28003195

[B16] Cameron M. Williams H. E. Cannane A. (2004). Improved gapped alignment in BLAST. *IEEE ACM Trans. Comput. Biol. Bioinform.* 1 116–129. 10.1109/TCBB.2004.32 17048387

[B17] Che J. Shi J. Gao Z. Zhang Y. (2016). Transcriptome analysis reveals the genetic basis of the resveratrol biosynthesis pathway in an endophytic fungus (*Alternaria* sp. MG1) isolated from *Vitis vinifera*. *Front. Microbiol.* 7:1257. 10.3389/fmicb.2016.01257 27588016 PMC4988973

[B18] Chen H. Liu L. Wang L. Wang S. Somta P. Cheng X. (2015). Development and validation of EST-SSR markers from the transcriptome of adzuki bean (*Vigna angularis*). *PLoS One* 10:e0131939. 10.1371/journal.pone.0131939 26146990 PMC4492930

[B19] Conesa A. Götz S. García-Gómez J. M. Terol J. Talón M. Robles M. (2005). Blast2GO: a universal tool for annotation, visualization and analysis in functional genomics research. *Bioinformatics* 21 3674–3676. 10.1093/bioinformatics/bti610 16081474

[B20] Cooke D. E. L. Lees A. K. (2004). Markers, old and new, for examining *Phytophthora infestans* diversity. *Plant Pathol.* 53 692–704. 10.1111/j.1365-3059.2004.01104.x

[B21] Estabrook G. F. Landrum L. (1975). A simple test for the possible simultaneous evolutionary divergence of two amino acid positions. *Taxon* 24 609–613. 10.2307/1220730

[B22] Evanno G. Regnaut S. Goudet J. (2005). Detecting the number of clusters of individuals using the software STRUCTURE: a simulation study. *Mol. Ecol.* 14 2611–2620. 10.1111/j.1365-294X.2005.02553.x 15969739

[B23] Ewens W. J. (1972). The sampling theory of selectively neutral alleles. *Theor. Popul. Biol.* 3 87–112. 10.1016/0040-5809(72)90035-4 4667078

[B24] Excoffier L. Laval G. Schneider S. (2005). Arlequin (version 3.0): an integrated software package for population genetics data analysis. *Evol. Bioinform. Online* 1 47–50. 10.1177/117693430500100003 19325852 PMC2658868

[B25] Gannibal P. B. Klemsdal S. S. Levitin M. M. (2007). AFLP analysis of Russian *Alternaria tenuissima*, populations from wheat kernels and other hosts. *Eur. J. Plant Pathol.* 119 175–182. 10.1007/s10658-007-9159-z

[B26] Glass N. L. Donaldson G. C. (1995). Development of primer sets designed for use with the PCR to amplify conserved genes from filamentous ascomycetes. *Appl. Environ. Microbiol.* 61 1323–1330. 7747954 10.1128/aem.61.4.1323-1330.1995 PMC167388

[B27] Grünwald N. J. Goodwin S. B. Milgroom M. G. Fry W. E. (2003). Analysis of genotypic diversity data for populations of microorganisms. *Phytopathology* 93 738–746. 10.1094/PHYTO.2003.93.6.738 18943061

[B28] Haas B. J. Papanicolaou A. Yassour M. Grabherr M. Blood P. D. Bowden J. (2013). *De novo* transcript sequence reconstruction from RNA-seq using the Trinity platform for reference generation and analysis. *Nat. Protoc.* 8 1494–1512. 10.1038/nprot.2013.084 23845962 PMC3875132

[B29] Hemmati R. Javan-Nikkhah M. Linde C. C. (2009). Population genetic structure of *Sclerotinia sclerotiorum* on canola in Iran. *Eur. J. Plant Pathol.* 125:617 10.1007/s10658-009-9510-7

[B30] Hu J. Chen C. Peever T. Dang H. Lawrence C. Mitchell T. (2012). Genomic characterization of the conditionally dispensable chromosome in *Alternaria arborescens* provides evidence for horizontal gene transfer. *BMC Genomics* 13:171. 10.1186/1471-2164-13-171 22559316 PMC3443068

[B31] Jakobsson M. Rosenberg N. A. (2007). CLUMPP: a cluster matching and permutation program for dealing with label switching and multimodality in analysis of population structure. *Bioinformatics* 23 1801–1806. 10.1093/bioinformatics/btm233 17485429

[B32] Kreis R. A. Dillard H. R. Smart C. D. (2016). Population diversity and sensitivity to azoxystrobin of *Alternaria brassicicola* in New York State. *Plant Dis.* 100 2422–2426. 10.1094/PDIS-03-16-0414-RE 30686174

[B33] Lee S. B. Taylor J. W. (1990). “Isolation of DNA from fungal mycelium and single spores,” in *PCR Protocols. A Guide to Methods and Applications* eds Innis M. A. Gelfand D. H. Sninsky J. J. White T. J. (San Diego, CA: Academic Press) 282–287.

[B34] Li H. Handsaker B. Wysoker A. Fennell T. Ruan J. Homer N. (2009). The sequence alignment/map format and SAMtools. *Bioinformatics* 25 2078–2079. 10.1093/bioinformatics/btp352 19505943 PMC2723002

[B35] Li M. Y. Wang F. Jiang Q. Ma J. Xiong A. S. (2014). Identification of SSRs and differentially expressed genes in two cultivars of celery (*Apium graveolens* L.) by deep transcriptome sequencing. *Hortic. Res.* 1:10. 10.1038/hortres.2014.10 26504532 PMC4596314

[B36] Linde C. C. Liles J. A. Thrall P. H. (2010). Expansion of genetic diversity in randomly mating founder populations of *Alternaria brassicicola* infecting *Cakile maritima* in Australia. *Appl. Environ. Microbiol.* 76 1946–1954. 10.1128/AEM.01594-09 20097819 PMC2837991

[B37] Linde C. C. Zala M. Mc Donald B. A. (2009). Molecular evidence for recent founder populations and human–mediated migration in the barley scald pathogen *Rhynchosporium secalis*. *Mol. Phylogenet. Evol.* 51 454–464. 10.1016/j.ympev.2009.03.002 19289174

[B38] Malik T. Hussain S. Hassan S. (1991). Studies on seedborne mycoflora of tomato in the NWFP. *Sarhad J. Agric.* 7 773–777.

[B39] McDonald B. A. (1997). The population genetics of fungi: tools and techniques. *Phytopathology* 87 448–453. 10.1094/PHYTO.1997.87.4.448 18945126

[B40] McDonald B. A. Linde C. (2002). The population genetics of plant pathogens and breeding strategies for durable resistance. *Euphytica* 124 163–180. 10.1023/A:1015678432355

[B41] Meng J. W. He D. C. Zhu W. Yang L. N. Wu E. Xie J. H. (2018). Human-mediated gene flow contributes to metapopulation genetic structure of the pathogenic fungus *Alternaria alternata* from Potato. *Front. Plant Sci.* 9:198. 10.3389/fpls.2018.00198 29497439 PMC5818430

[B42] Meng J. W. Zhu W. He M. H. Wu E. J. Yang L. N. Shang L. P. (2015). High genotype diversity and lack of isolation by distance in the *Alternaria solani* populations from China. *Plant Pathol.* 64 434–441. 10.1111/ppa.12275

[B43] Milgroom M. G. (1996). Recombination and the multilocus structure of fungal populations. *Annu. Rev. Phytopathol.* 34 457–477. 10.1146/annurev.phyto.34.1.457 15012552

[B44] Miller N. J. Birley A. J. Overall A. D. J. Tatchell G. M. (2003). Population genetic structure of the lettuce root aphid, *Pemphigus bursarius* (L.), in relation to geographic distance, gene flow and host plant usage. *Heredity* 91 217–223. 10.1038/sj.hdy.6800331 12939621

[B45] Morris P. F. Connolly M. S. Clair D. A. S. (2000). Genetic diversity of *Alternaria alternata*, isolated from tomato in California assessed using RAPDs. *Mycol. Res.* 104 286–292. 10.1017/S0953756299008758

[B46] Nei M. (1973). Analysis of gene diversity in subdivided populations. *Proc. Natl. Acad. Sci. U.S.A.* 70 3321–3323. 10.1073/pnas.70.12.3321 4519626 PMC427228

[B47] Nei M. (1978). Estimation of average heterozygosity and genetic distance from a small number of individuals. *Genetics* 89 583–590. 17248844 10.1093/genetics/89.3.583 PMC1213855

[B48] Nei M. Chesser R. K. (1983). Estimation of fixation indices and gene diversities. *Ann. Hum. Genet.* 47 253–259. 10.1111/j.1469-1809.1983.tb00993.x 6614868

[B49] Nguyen H. D. Lewis C. T. Lévesque C. A. Gräfenhan T. (2016). Draft genome sequence of *Alternaria alternata* ATCC 34957. *Genome Announc.* 4:e01554-15. 10.1128/genomeA.01554-15 26769939 PMC4714121

[B50] Parker I. M. Gilbert G. S. (2004). The evolutionary ecology of novel plant-pathogen interactions. *Annu. Rev. Ecol. Evol. Syst.* 35 675–700. 10.1146/annurev.ecolsys.34.011802.132339

[B51] Peakall R. Smouse P. E. (2006). GENALEX 6: genetic analysis in excel. Population genetic software for teaching and research. *Mol. Ecol. Notes* 6 288–295. 10.1111/j.1471-8286.2005.01155.x 22820204 PMC3463245

[B52] Piotrowska M. J. Ennos R. A. Fountaine J. M. Burnett F. J. Kaczmarek M. Hoebe P. N. (2016). Development and use of microsatellite markers to study diversity, reproduction and population genetic structure of the cereal pathogen *Ramularia collo-cygni*. *Fungal Genet. Biol.* 87 64–71. 10.1016/j.fgb.2016.01.007 26806723

[B53] Pritchard J. K. Stephens M. Donnelly P. (2000). Inference of population structure using multilocus genotype data. *Genetics* 155 945–959. 10835412 10.1093/genetics/155.2.945 PMC1461096

[B54] Rahman M. Z. Honda Y. Islam S. Z. Muroguchi N. Arase S. (2002). Leaf spot disease of broad bean (*Vicia faba* L.) caused by *Alternaria tenuissima*-a new disease in Japan. *J. Gen. Plant Pathol.* 68 31–37. 10.1007/PL00013049

[B55] Raymond M. Rousset F. (1995). GENEPOP (version 1.2): population genetics software for exact tests and ecumenicism. *J. Hered.* 86 248–249. 10.1093/oxfordjournals.jhered.a111573

[B56] Rosenberg N. A. (2004). DISTRUCT: a program for the graphical display of population structure. *Mol. Ecol. Notes* 4 137–138. 10.1046/j.1471-8286.2003.00566.x

[B57] Santha Lakshmi Prasad M. Sujatha M. Chander Rao S. (2009). Analysis of cultural and genetic diversity in *Alternaria helianthi* and determination of pathogenic variability using wild *Helianthus* species. *J. Phytopathol.* 157 609–617. 10.1111/j.1439-0434.2009.01542.x

[B58] Stewart J. E. Kawabe M. Abdo Z. Arie T. Peever T. L. (2011). Contrasting codon usage patterns and purifying selection at the mating locus in putatively asexual Alternaria fungal species. *PLoS One* 6:e20083. 10.1371/journal.pone.0020083 21625561 PMC3098265

[B59] Tsui C. K. M. Roe A. D. El-Kassaby Y. A. Rice A. V. Alamouti S. M. Sperling F. A. H. (2012). Population structure and migration pattern of a conifer pathogen, *Grosmannia clavigera*, as influenced by its symbiont, the mountain pine beetle. *Mol. Ecol.* 21 71–86. 10.1111/j.1365-294X.2011.05366.x 22118059

[B60] Van Der Waals J. E. Korsten L. Slippers B. (2004). Genetic diversity among *Alternaria solani* isolates from potatoes in South Africa. *Plant Dis.* 88 959–964. 10.1094/PDIS.2004.88.9.959 30812248

[B61] Wang T. Y. Zhao J. Sun P. Wu X. H. (2014). Characterization of *Alternaria* species associated with leaf blight of sunflower in China. *Eur. J. Plant Pathol.* 140 301–315. 10.1007/s10658-014-0464-z

[B62] Watterson G. (1978). The homozygosity test of neutrality. *Genetics* 88 405–417. 17248803 10.1093/genetics/88.2.405 PMC1213809

[B63] Woudenberg J. H. C. Seidl M. F. Groenewald J. Z. De Vries M. Stielow J. B. Thomma B. P. H. J. (2015). Alternaria section *Alternaria*: species, *formae speciales* or pathotypes? *Stud. Mycol.* 82 1–21. 10.1016/j.simyco.2015.07.001 26951037 PMC4774270

[B64] Wright E. R. Rivera M. C. Esperón J. Cheheid A. Rodríguez Codazzi A. (2004). *Alternaria* leaf spot, twig blight, and fruit rot of highbush blueberry in Argentina. *Plant Dis.* 88 1383–1383. 10.1094/PDIS.2004.88.12.1383B 30795208

[B65] Wu Z. J. Li X. H. Liu Z. W. Xu Z. S. Zhuang J. (2014). *De novo* assembly and transcriptome characterization: novel insights into catechins biosynthesis in *Camellia sinensis*. *BMC Plant Biol.* 14:277. 10.1186/s12870-014-0277-4 25316555 PMC4203915

[B66] Yeh F. C. Yang R. Boyle T. B. J. Ye Z. Mao J. X. (1997). *POPGENE, The User-Friendly Shareware for Population Genetic Analysis*. Edmonton, AB: University of Alberta.

[B67] Zhan J. McDonald B. A. (2013). Experimental measures of pathogen competition and relative fitness. *Annu. Rev. Phytopathol.* 51 131–153. 10.1146/annurev-phyto-082712-102302 23767846

[B68] Zhan J. Pettway R. E. McDonald B. A. (2003). The global genetic structure of the wheat pathogen *Mycosphaerella graminicola* is characterized by high nuclear diversity, low mitochondrial diversity, regular recombination, and gene flow. *Fungal Genet. Biol.* 38 286–297. 10.1016/S0187-1845(02)00538-8 12684018

[B69] Zhang S. Chen W. Xin L. Gao Z. Hou Y. Yu X. (2014). Genomic variants of genes associated with three horticultural traits in apple revealed by genome re-sequencing. *Hortic. Res.* 1:14045. 10.1038/hortres.2014.45 26504548 PMC4596325

[B70] Zhao J. Bao S. W. Ma G. P. Wu X. H. (2016a). Characterization of *Alternaria* species associated with watermelon leaf blight in Beijing municipality of China. *J. Plant Pathol.* 98 135–138.

[B71] Zhao J. Bao S. W. Ma G. P. Wu X. H. (2016b). Characterization of *Alternaria* species associated with muskmelon foliar diseases in Beijing municipality of China. *J. Gen. Plant Pathol.* 82 29–32. 10.1007/s10327-015-0631-x

[B72] Zheng H. H. Zhao J. Wang T. Y. Wu X. H. (2015). Characterization of Alternaria species associated with potato foliar diseases in China. *Plant Pathol.* 64 425–433. 10.1111/ppa.12274

[B73] Zheng X. Pan C. Diao Y. You Y. Yang C. Hu Z. (2013). Development of microsatellite markers by transcriptome sequencing in two species of *Amorphophallus* (Araceae). *BMC Genomics* 14:490. 10.1186/1471-2164-14-490 23870214 PMC3737116

