# Supplementary figures and images for: The Population Genetics of Alternaria tenuissima in Four Regions of China as Determined by Microsatellite Markers Obtained by Transcriptome Sequencing
Source: Front Microbiol. 2018 Dec 3;9:2904. doi: 10.3389/fmicb.2018.02904 (PMC6287023; doi:10.3389/fmicb.2018.02904)

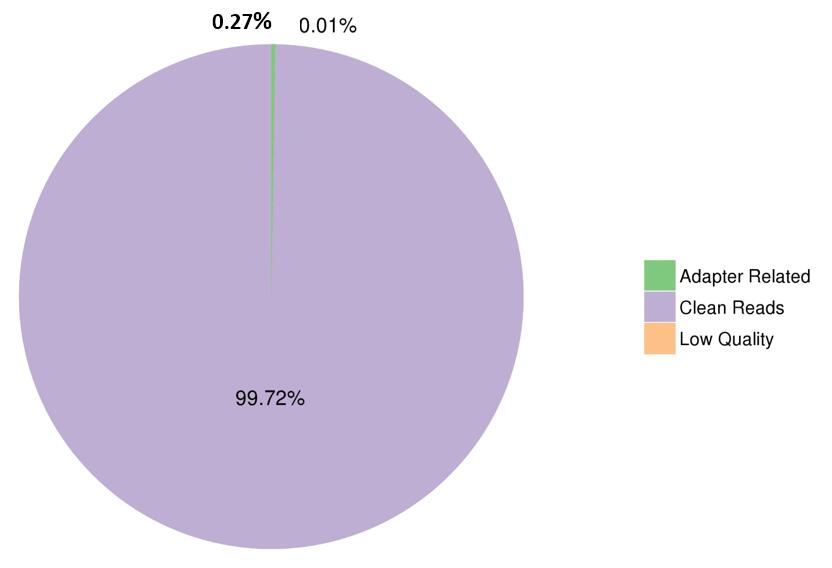

Supplement: FIGURE S1 — The percentage of clean reads, adapter related reads, and low quality reads. [file Image_1.JPEG]

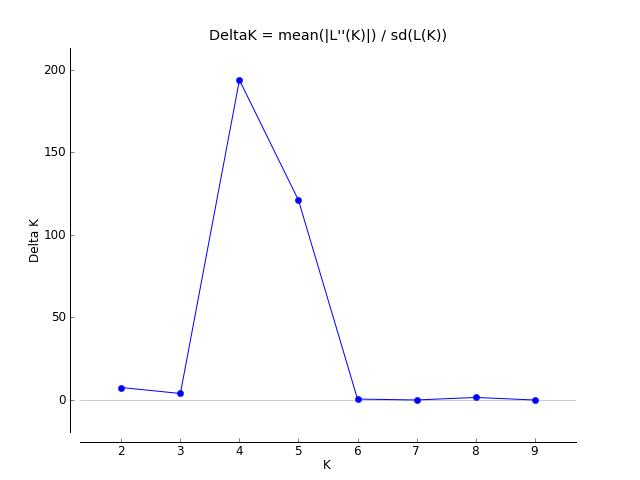

Supplement: FIGURE S2 — The estimated Delta K (K) for number of clusters ranging from 2 to 10 in STRUCTURE analysis. [file Image_2.JPEG]

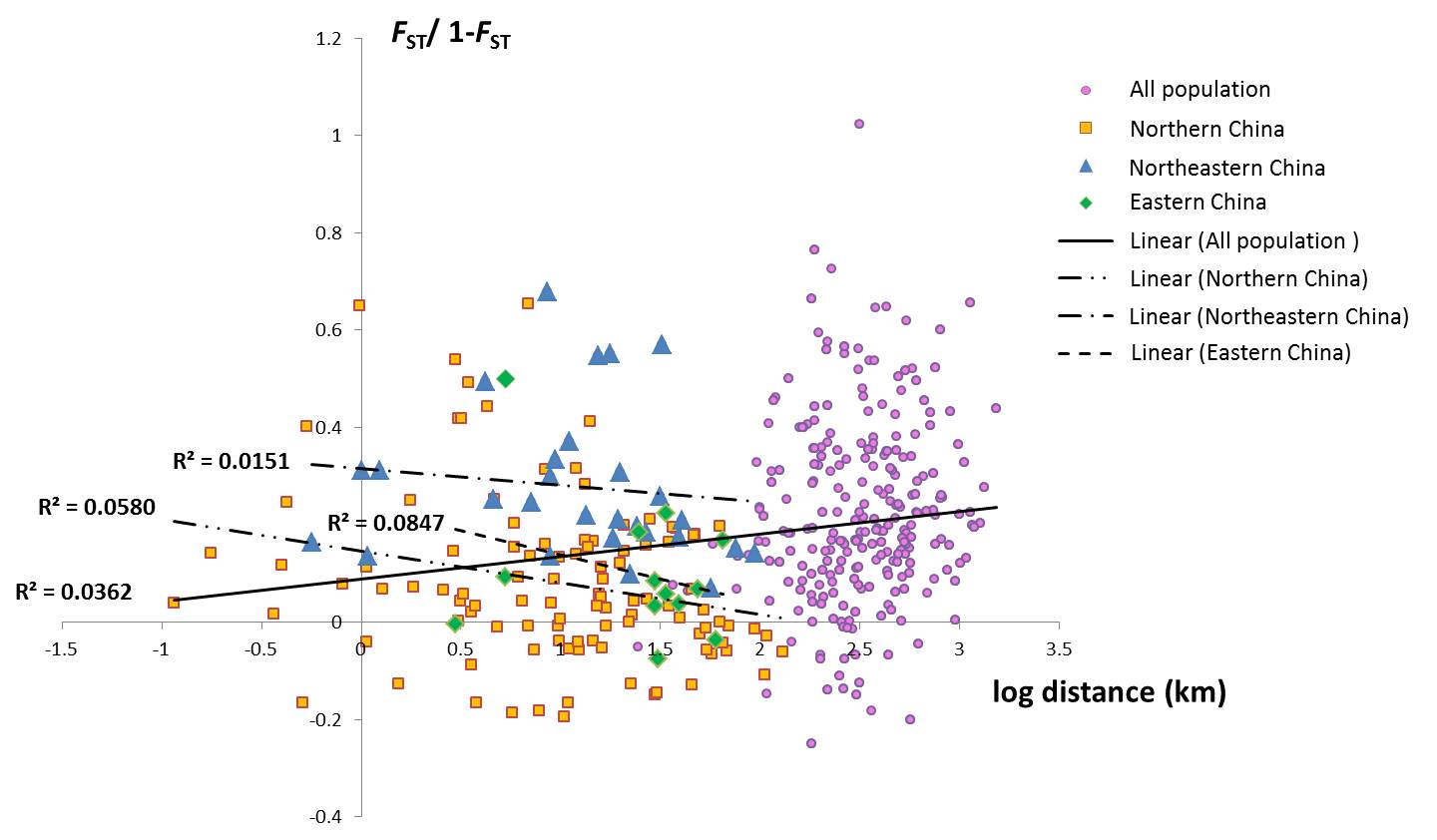

Supplement: FIGURE S3 — Plot of isolation by distance for the entire population, the geographic region Northern China, the geographic region Northeastern China, and the geographic region Eastern China. [file Image_3.JPEG]
